# Supplementary material for: Novel Driver Strength Index highlights important cancer genes in TCGA PanCanAtlas patients
Source: PeerJ. 2022 Aug 11;10:e13860. doi: 10.7717/peerj.13860 (PMC9375969; doi:10.7717/peerj.13860)
Supplement: Supplemental Information 2 [file peerj-10-13860-s002.zip › COHORTS/genes plots/detailed/2021_8_16_14_9_distribution_events_detailed_females_14.pdf]

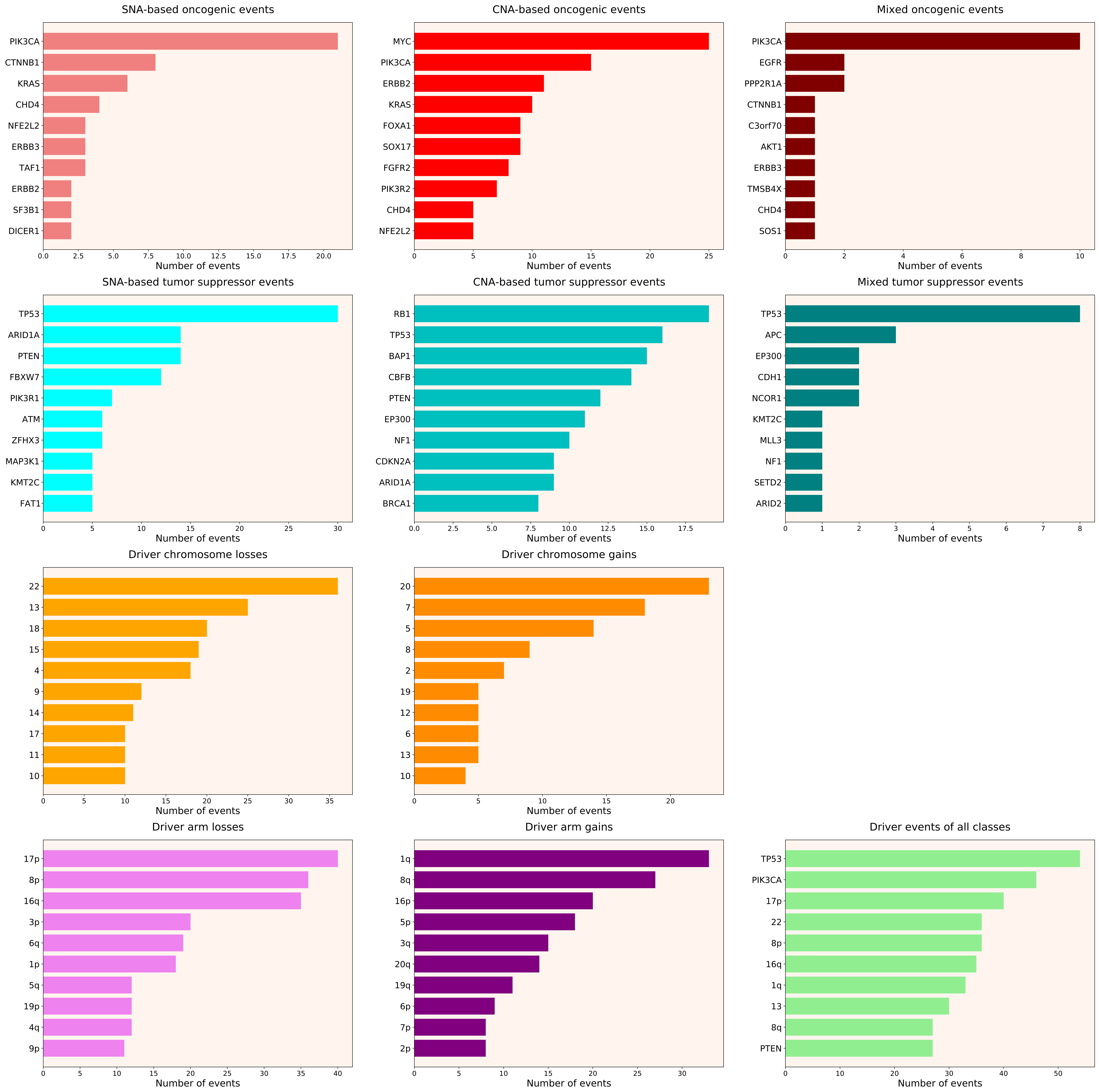
SNA-based oncogenic events
PIK3CA
CTNNB1
KRAS
CHD4
NFE2L2
ERBB3
TAF1
ERBB2
SF3B1
DICER1
0.0 2.5 5.0 7.5 10.0 12.5 15.0 17.5 20.0
Number of events
CNA-based oncogenic events
MYC
PIK3CA
ERBB2
KRAS
FOXA1
SOX17
FGFR2
PIK3R2
CHD4
NFE2L2
0 5 10 15 20 25
Number of events
Mixed oncogenic events
PIK3CA
EGFR
PPP2R1A
CTNNB1
C3orf70
AKT1
ERBB3
TMSB4X
CHD4
SOS1
0 2 4 6 8 10
Number of events
SNA-based tumor suppressor events
TP53
ARID1A
PTEN
FBXW7
PIK3R1
ATM
ZFHX3
MAP3K1
KMT2C
FAT1
0 5 10 15 20 25 30
Number of events
CNA-based tumor suppressor events
RB1
TP53
BAP1
CBFB
PTEN
EP300
NF1
CDKN2A
ARID1A
BRCA1
0.0 2.5 5.0 7.5 10.0 12.5 15.0 17.5
Number of events
Mixed tumor suppressor events
TP53
APC
EP300
CDH1
NCOR1
KMT2C
MLL3
NF1
SETD2
ARID2
0 1 2 3 4 5 6 7 8
Number of events
Driver chromosome losses
22
13
18
15
4
9
14
17
11
10
0 5 10 15 20 25 30 35
Number of events
Driver chromosome gains
20
7
5
8
2
19
12
6
13
10
0 5 10 15 20
Number of events
Driver arm losses
17p
8p
16q
3p
6q
1p
5q
19p
4q
9p
0 5 10 15 20 25 30 35 40
Number of events
Driver arm gains
1q
8q
16p
5p
3q
20q
19q
6p
7p
2p
0 5 10 15 20 25 30
Number of events
Driver events of all classes
TP53
PIK3CA
17p
22
8p
16q
1q
13
8q
PTEN
0 10 20 30 40 50
Number of events
